# Purification of Logic-Qubit Entanglement

**DOI:** 10.1038/srep28813

**Published:** 2016-07-05

**Authors:** Lan Zhou, Yu-Bo Sheng

**Affiliations:** 1 College of Mathematics & Physics, Nanjing University of Posts and Telecommunications, Nanjing, 210003, China; 2 Key Lab of Broadband Wireless Communication and Sensor Network Technology, Nanjing University of Posts and Telecommunications, Ministry of Education, Nanjing, 210003, China

## Abstract

Recently, the logic-qubit entanglement shows its potential application in future quantum communication and quantum network. However, the entanglement will suffer from the noise and decoherence. In this paper, we will investigate the first entanglement purification protocol for logic-qubit entanglement. We show that both the bit-flip error and phase-flip error in logic-qubit entanglement can be well purified. Moreover, the bit-flip error in physical-qubit entanglement can be completely corrected. The phase-flip in physical-qubit entanglement error equals to the bit-flip error in logic-qubit entanglement, which can also be purified. This entanglement purification protocol may provide some potential applications in future quantum communication and quantum network.

Entanglement plays an important role in quantum information areas. Quantum teleportation^1^, quantum key distribution (QKD)^2^, quantum secure direct communication (QSDC)^3^,^4^,^5^, quantum repeaters^6^,^7^ and other important quantum information protocols^8^,^9^,^10^,^11^,^12^, all need entanglement. Before starting the quantum communication protocol, the parties should set up the maximally entanglement channel first. Usually, they create the entanglement locally and distribute the entangled state to distant locations in fiber or free space. Noise is one of the main obstacles in entanglement distribution. It will degrade the entanglement. The degraded entanglement will decrease the efficiency of the communication and even make the quantum communication insecure.

Entanglement purification is to distill the high quality entangled states from the low quality entangled states with local operation and classical communications (LOCC). In 1996, Bennett *et al*. proposed the concept of entanglement purification^13^. Subsequently, there are many efficient entanglement purification protocols (EPPs) proposed^14^,^15^,^16^,^17^,^18^,^19^,^20^,^21^,^22^,^23^,^24^,^25^,^26^,^27^,^28^,^29^,^30^,^31^,^32^,^33^,^34^,^35^,^36^. For example, in 2001, Pan *et al*. described a feasible EPP with linear optics^16^. In 2008, Sheng *et al*. described an EPP which can be repeated to obtain a higher fidelity^18^. In 2010, the first deterministic EPP was proposed^19^. The deterministic EPPs^19^,^20^,^21^,^22^ are quite different from the previous EPPs^13^,^14^,^15^,^16^,^17^,^18^. The deterministic EPP can obtain the maximally entangled state in one or two steps with the 100% success probability, in principle, while the previous EPPs are to increase the fidelity of mixed states by repeating the EPPs. In 2014, the EPP for hyperentanglement was presented^27^. Recent researches showed that the entanglement purification can be used to benefit the blind quantum computation^29^. There are also some important EPPs for solid systems, such as the EPP for spins^30^, short chains of atoms^32^,^33^, ionic states^36^, and so on.

The EPPs described above all focus on the entanglement encoded in the physical qubit directly, for existing quantum communication protocols are usually based on the physical-qubit entanglement. Recently, Froẅis and Dür investigated a new type of entanglement, named concatenated Greenberger-Horne-Zeilinger (C-GHZ) state^37^. The C-GHZ state can be written as^37^,^38^,^39^,^40^,^41^,^42^,^43^,^44^,^45^,^46^,^47^,^48^


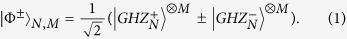


Here *M* is the number of the logic qubit and *N* is the number of the physical qubit in each logic qubit. Each logic qubit is a physical GHZ state of the form


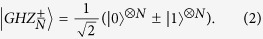


In 2014, Lu *et al*. realized the first experiment of logic-qubit entanglement in linear optics^42^. In 2015, Sheng and Zhou described the first logic Bell-state analysis^43^. They showed that we can perform the logic-qubit entanglement swapping and it is possible to perform the long-distance quantum communication based on logic-qubit entanglement^44^,^45^,^46^. These theory and experiment researches may provide an important avenue that the large-scale quantum networks and the quantum communication may be based on logic-qubit entanglement in future.

Though many EPPs were proposed and discussed, none protocol discusses the purification of logic-qubit entanglement. In this paper, we will investigate the first model of entanglement purification for logic-qubit entanglement. We show that both the bit-flip error and phase-flip error in logic-qubit entanglement can be well purified. With the help of controlled-not (CNOT) gate, the EPP of logic-qubit entanglement can be simplified to the EPP of physical-qubit entanglement, which can be easily purified in the next step. Moreover, we also show that if a bit-flip error occurs in one of a physical-qubit entanglement locally, it can be well corrected. Moreover, the phase-flip error in one of a physical-qubit entanglement equals to the bit-flip error in the logic qubit entanglement, which can be well purified.

This paper is organized as follows: In Sec. II, we explain the purification for logic qubit error. In Sec. III, we describe the purification for physical qubit error. In Sec. IV, we present a discussion and conclusion.

## Results

Suppose that Alice and Bob share the maximally logic Bell state 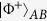. The four logic Bell states can be described as


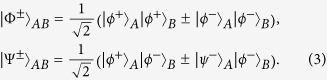


Here |*ϕ*^±^〉 and |*ψ*^±^〉 are four physical Bell states of the form


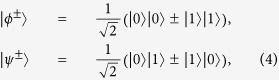


with |0〉 and |1〉 being the physical qubits, respectively. From Eq. (3), the Bell states |*ϕ*^+^〉 and |*ϕ*^−^〉 can be regarded as the logic qubit  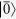 and  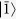, respectively. |Φ^+^〉_*AB*_ essentially is the state with *M* = *N* = 2 in Eq. (1). If a bit-flip error occurs on the logic qubit with the probability of 1 − *F*, |Φ^+^〉_*AB*_ will become |Ψ^+^〉_*AB*_. The whole mixed state can be described as


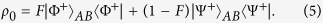


**Figure 1:**
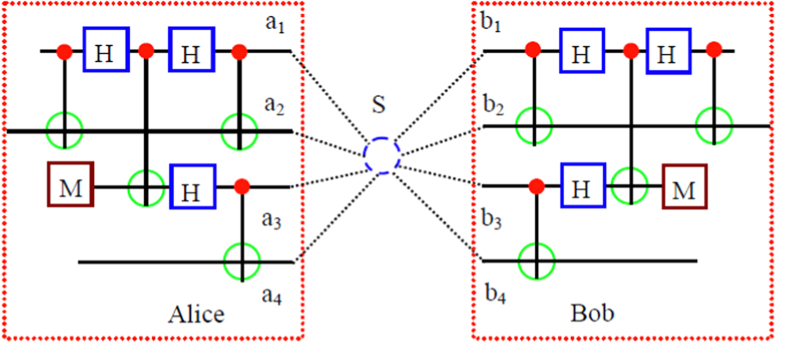
Schematic diagram of the purification of logic Bell-state analysis. *H* represents the Hadamard operation and *M* represents the measurement in the basis {|0〉, |1〉}.

As shown in Fig. 1, Alice and Bob share two copies of mixed states, named *ρ*_*1*_ and *ρ*_*2*_, distributed from the entanglement source *S*. State *ρ*_*1*_ is in the spatial modes *a*_*1*_, *a*_*2*_, *b*_*1*_ and *b*_*2*_ and state *ρ*_*2*_ is in the spatial modes *a*_*3*_, *a*_*4*_, *b*_*3*_ and *b*_*4*_, respectively. The whole system *ρ*_1_ ⊗ *ρ*_2_ can be described as follows. With the probability of *F*^2^, it is in the state |Φ^+^〉_*A*1*B*1_ ⊗ |Φ^+^〉_*A*2*B*2_. With the equal probability of *F*(1 − *F*), it is in the states |Φ^+^〉_*A*1*B*1_ ⊗ |Ψ^+^〉_*A*2*B*2_ or |Ψ^+^〉_*A*1*B*1_ ⊗ |Φ^+^〉_*A*2*B*2_. With the probability of (1 − *F*)^2^, it is in the state |Ψ^+^〉_*A*1*B*1_ ⊗ |Ψ^+^〉_*A*2*B*2_. Here states |Φ^+^〉_*A*1*B*1_ and |Ψ^+^〉_*A*1*B*1_ are the components in *ρ*_*1*_ and |Φ^+^〉_*A*2*B*2_ and |Ψ^+^〉_*A*2*B*2_ are the components in *ρ*_*2*_, respectively.

We first discuss the item |Φ^+^〉_*A*1*B*1_ ⊗ |Φ^+^〉_*A*2*B*2_. It can be written as


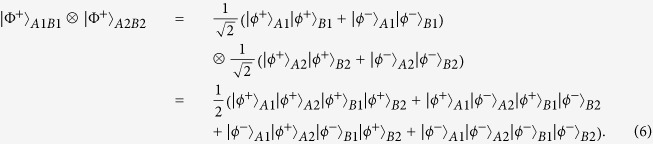


From Fig. 1, they let all qubits pass through the controlled-not (CNOT) gate. State |*ϕ*^+^〉_*A*1_ in spatial modes *a*_*1*_, *a*_*2*_ will become


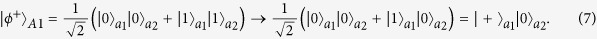


State |*ϕ*^−^〉_*A*1_ in spatial modes *a*_*1*_, *a*_*2*_ will become


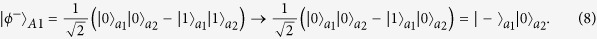


Here 
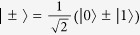
. After passing through the CNOT gates and Hadamard gates, with the probability of *F*^2^, state in Eq. (6) can be evolved as


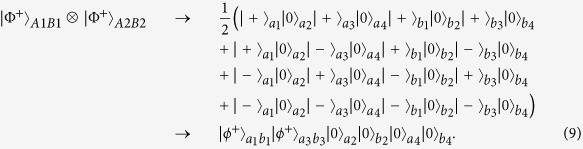


Following the same principle, with the probability of *F*(1 − *F*), state |Φ^+^〉_*A*1*B*1_ ⊗ |Ψ^+^〉_*A*2*B*2_ can be evolved as


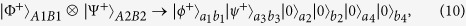


and state |Ψ^+^〉_*A*1*B*1_ ⊗ |Φ^+^〉_*A*2*B*2_ can be evolved as


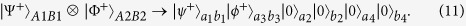


With the probability of (1 − *F*)^2^, state |Ψ^+^〉_*A*1*B*1_ ⊗ |Ψ^+^〉_*A*2*B*2_ can be evolved as


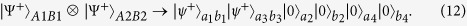


Here 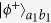, 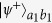,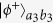 and 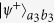 are the physical Bell states described in Eq. (4) in spatial modes *a*_*1*_*b*_*1*_, *a*_*3*_*b*_*3*_, respectively. Interestingly, from Eq. (9) to Eq. (12), the qubits in spatial modes *a*_*2*_, *b*_*2*_, *a*_*4*_ and *b*_*4*_ disentangle with the other qubits. The purification of logic Bell state can be transformed to the purification of the physical Bell state in spatial modes *a*_*1*_, *b*_*1*_, *a*_*3*_ and *b*_*3*_. Briefly speaking, as shown in Fig. 1, they let the qubits in *a*_*1*_, *b*_*1*_, *a*_*3*_ and *b*_*3*_ modes pass through the CNOT gates for a second time. The CNOT gates will make the state^13^


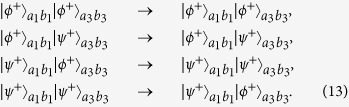


Subsequently, Alice and Bob measure their qubits in spatial modes *a*_*3*_ and *b*_*3*_ in {0, 1} basis, respectively. With classical communication, if the measurement results are the same, the purification is successful. Otherwise, if the measurement results are different, the purification is a failure. From Eq. (13), if it is successful, they will obtain 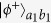, with the probability of *F*^2^, and  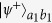 will the probability of (1 − *F*)^2^. In this way, they obtain a new mixed state


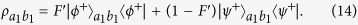


Here


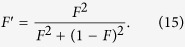


If 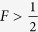, they can obtain *F*′ > *F*. State in Eq. (14) is the purified physical Bell state. The final step is to recover 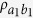 to logic Bell state. From Fig. 1, they perform the Hadamard operations on the qubits in spatial modes *a*_*1*_ and *b*_*1*_ *a*nd let four qubits in *a*_*1*_, *b*_*1*_, *a*_*2*_ *a*nd *b*_*2*_ p*a*ss through the CNOT gates, respectively. State  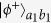 combined with 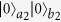 evolves as


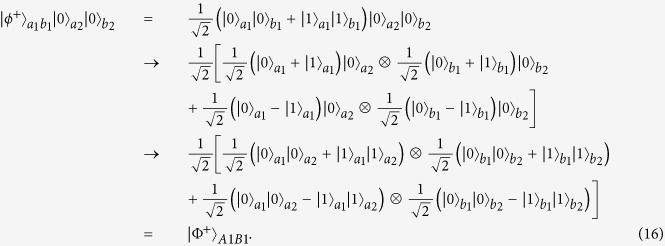


Following the same principle, state  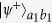 combined with  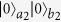 evolve to |Ψ^+^〉_*A*1*B*1_. Finally, they will obtain a new mixed state


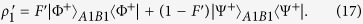


In this way, they have completed the purification.

On the other hand, if a phase-flip error occurs, it will make the state in Eq. (3) become


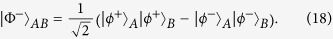


The whole mixed state can be written as


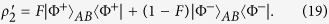


The mixed state in Eq. (19) can also be purified with the same principle. Briefly speaking, as shown in Fig. 1, they first choose two copies of the states in Eq. (19). After the qubits in spatial modes *a*_1_, *a*_2_, *b*_1_, *b*_2_, *a*_3_, *a*_4_, *b*_3_ and *b*_4_ passing through the CNOT gates and Hadamard gates, respectively. |Φ^+^〉_*A*1*B*1_ ⊗ |Φ^+^〉_*A*2*B*2_ will become 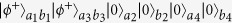, which is shown in Eq. (9). State |Φ^+^〉_*A*1*B*1_ ⊗ |Φ^−^〉_*A*2*B*2_ will become 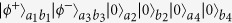. State |Φ^−^〉_*A*1*B*1_ ⊗ |Φ^+^〉_*A*2*B*2_ will become 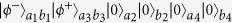, and state |Φ^−^〉_*A*1*B*1_ ⊗ |Φ^−^〉_*A*2*B*2_ will become 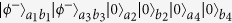. They only need to add the Hadamard operations on each qubit, which make |*ϕ*^+^〉 not change, and |*ϕ*^−^〉 become |*ψ*^+^〉. They essentially transform the phase-flip error to bit-flip error, which has the same form as described above. In this way, the phase-flip error in logic-qubit entanglement can also be purified.

So far, we have described the EPP for logic-qubit entanglement. Each logic qubit is encoded in a physical Bell state. It is straightforward to extend this approach to the logic-qubit entanglement with arbitrary physical GHZ state encoded in a logic qubit. Suppose that Alice and Bob share the state


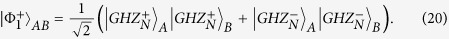


The noise makes the state become


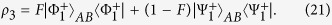


Here


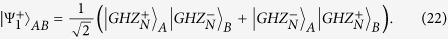


**Figure 2:**
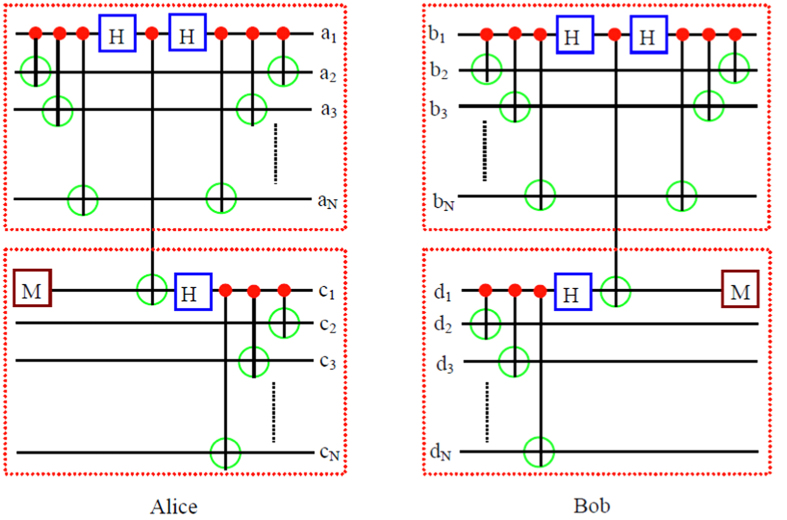
Schematic diagram of the EPP with each logic qubit being arbitrary GHZ state. On pair of mixed state *ρ*_*ab*_ is in the spatial modes *a*_*1*_, *b*_*1*_, *a*_*2*_, *b*_*2*_, ···, *a*_*N*_ and *b*_*N*_. The other copy of mixed state *ρ*_*cd*_ is in the spatial modes *c*_*1*_, *d*_*1*_, *c*_*2*_, *d*_*2*_, ···, *c*_*N*_ and *d*_*N*_.

As shown in Fig. 2, they first choose two copies of the mixed states with the same form of *ρ*_*3*_. One mixed state *ρ*_*ab*_ is in the spatial modes *a*_*1*_, *b*_*1*_, *a*_*2*_, *b*_*2*_, ···, *a*_*N*_, *b*_*N*_, and the other mixed state *ρ*_*cd*_ is in the spatial modes *c*_*1*_, *d*_*1*_, *c*_*2*_, *d*_*2*_, ···, *c*_*N*_, *d*_*N*_, respectively. We first discuss the mixed state *ρ*_*ab*_. After passing through the CNOT gates and Hadamard gates, with the probability of *F*, state  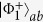 becomes


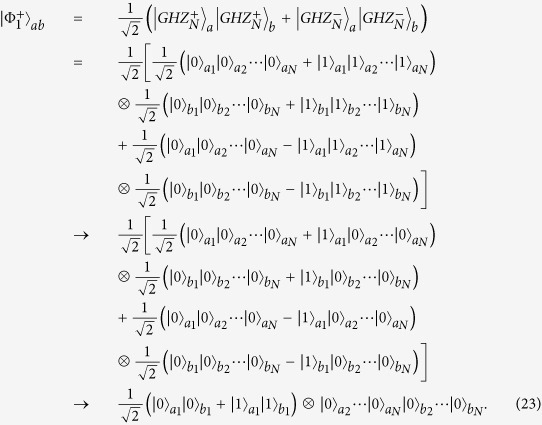


With the same principle, with the probability of 1 − *F*, state 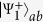 becomes


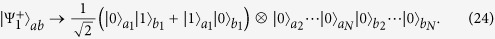


Similar to Eqs (23) and (24), after passing through the CNOT gates and Hadamard gates, state *ρ*_*cd*_ in the spatial modes *c*_*1*_, *d*_*1*_, *c*_*2*_, *d*_*2*_, ···, *c*_*N*_, *d*_*N*_ can also evolve as


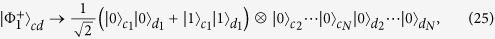


and


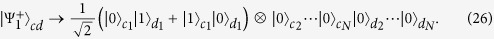


Here the subscripts *a*, *b*, *c* and *d* are the spatial modes as shown in Fig. 2. From Eqs (23) to (26), 46, 47, 48, by choosing two copies of mixed states *ρ*_*ab*_ and *ρ*_*cd*_, they can be simplified to the purification of the physical Bell state, which can be easily performed, similar to Eqs (9) to (12), 46, 47, 55. After they obtaining the purified high fidelity physical Bell state, the last step is also to recover the physical Bell state to arbitrary logic Bell state. They first perform the Hadamard operation on the qubits in *a*_1_ mode and *b*_1_ mode, respectively. Subsequently, they both let the *N* qubits pass through *N* − 1 CNOT gates, respectively. Finally, they can obtain a high fidelity of arbitrary logic-qubit entangled state.

On the other hand, if a phase-flip error occurs, it makes the state  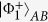 become  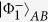, which can be written as


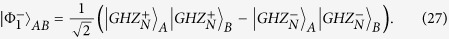


The mixed state can be written as


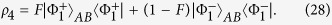


Interestingly, after passing through the CNOT gates and Hadamard gates, state  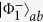 will become


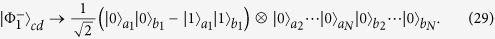


From Eqs (23), (28) and (29), we can find that the phase-flip error in the logic-qubit entanglement can be simplified into the phase-flip error of the physical-qubit Bell entanglement, which can be transformed to the bit-flip error and purified in the next step. In this way, they can purify arbitrary logic-qubit entanglement.

In above section, we showed that the bit-flip error and phase-flip error in the logic-qubit entanglement can be simplified into the bit-flip error and phase-flip error in the physical-qubit entanglement, respectively. Subsequently, the errors in the physical-qubit entanglement can be well purified with the similar approach as refs 13 and 14. Besides the errors in the logic-qubit entanglement, the single physical qubit can also suffer from the error. For example, as shown in Eq. (3), if a bit-flip error occurs in one of the physical qubits in the logic-qubit *A*, it makes |*ϕ*^+^〉_*A*_ become |*ψ*^+^〉_*A*_ and |*ϕ*^−^〉_*A*_ become |*ψ*^−^〉_*A*_, respectively. Therefore, it makes the state |Φ^+^〉_*AB*_ become


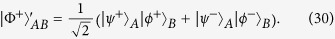


Compared with Eq. (3) and Eq. (30), we find that the error occurs locally. In this way, they only require to choose one copy of the mixed state to perform the error correction. They let the logic-qubit *A* pass through the CNOT gate. The qubit in *a*_1_ mode is the control qubit and the qubit in *a*_2_ mode is the target qubit. State in Eq. (3) will become


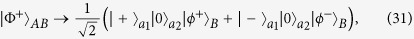


and state in Eq. (30) will become


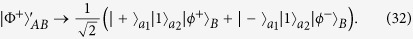


From Eqs (31) and (32), they only need to measure the physical qubit in *a*_*2*_ mode in the basis {0, 1}. If it becomes |1〉, it means that a bit-flip error occurs. If Alice and Bob exploit the quantum nondemolition (QND) measurement, which does not destroy the physical qubit, they are only required to perform a bit-flip operation to correct the bit-flip error. On the other hand, if the measurement is destructive, they can prepare another physical qubit |0〉 in *a*_2_ mode and perform the CNOT operation with the physical qubit *a*_1_ mode in logic qubit *A* to recover the whole state to |Φ^+^〉_*AB*_. If the bit-flip error occurs on the second logic qubit *B*, they can also completely correct it with the same principle.

If the logic qubit is *N*-particle GHZ state, a bit-flip error on the logic-qubit *A* will make the state become


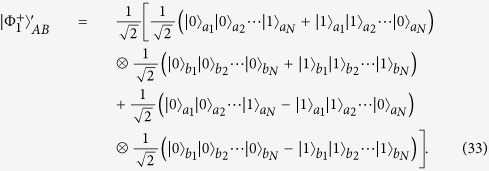


They let the particles in *a*_1_, *a*_2_, ···, *a*_*N*_ modes pass through the *N* − 1 CNOT gates. In each CNOT gate, particle in *a*_1_ mode is the control qubit and the other is the target qubit. It makes the state 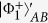 become


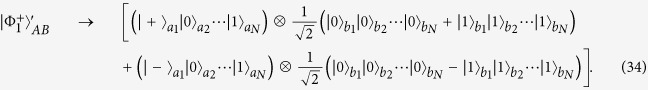


From Eq. (34), by measuring the physical qubit in *a*_*N*_ mode in the basis {0, 1}, if it becomes |1〉, it means that a bit-flip error occurs. Following the same principle, it can be completely corrected.

On the other hand, if a phase-flip error occurs on the logic qubit *A*, which makes |*ϕ*^+^〉 ↔ |*ϕ*^−^〉. The state 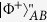 with a phase-flip error in logic qubit *A* can be written as


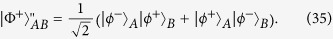


Interestingly, from Eq. (35), we find that the phase-flip error in the two physical qubits essentially equals to the logic bit-flip error as shown in Eq. (3). In this way, we have completely explained our EPP.

## Discussion

In traditional EPPs for physical-qubit entanglement^13^,^14^, they should purify two kinds of errors. The one is the bit-flip error and the other is the phase-flip error. Using the CNOT gate, the bit-flip error can be purified directly. The phase-flip error can be transformed to the bit-flip error and be purified in the next step. In our EPP, we show that the logic-qubit entanglement may contain four kinds of errors. The bit-flip error and phase-flip error occur in the logic-qubit entanglement and physical-qubit entanglement, respectively. From our description, the bit-flip error and phase-flip error in logic-qubit entanglement can be simplified to the bit-flip error and phase-flip error in physical-qubit entanglement, which can be purified with the previous approach in the next step. On the other hand, if a bit-flip error occurs in one logic qubit, the error can be completely corrected locally. Moreover, if a phase-flip error occurs in one logic qubit, we find that it equals to the bit-flip error in the logic-qubit entanglement. In this way, all errors can be purified. In our EPP, the key element to realize the protocol is the CNOT gate. There are some important progresses in construction of the CNOT gate, which shows that it is possible to realize the deterministic CNOT gate in future experiment^49^,^50^,^51^,^52^,^53^. For example, with the help cross-Kerr nonlinearity, Nemoto *et al*. and Lin *et al*. described a near deterministic CNOT gate for polarization photons, respectively^49^,^50^. Recently, Wei and Deng designed a deterministic CNOT gate on two photonic qubits by two single-photon input-output processes and the readout on an electron-medium spin confined in an optical resonant microcavity^51^. The deterministic CNOT gate for spins^52^, electron-spin qubits assisted by diamond nitrogen-vacancy centers inside cavities were also discussed^53^. Recently, the group of Du present a very important progress of experiment of fault-tolerant universal quantum gates with solid-state spins under ambient conditions^54^. They realized a universal set of logic gates in a nitrogen-vacancy center in diamond with an average single-qubit gate fidelity of 0.999952 and two-qubit (CNOT) gate fidelity of 0.992. Such high fidelity CNOT gate shows that it is feasible to realize this EPP in future.

In conclusion, we have described the first EPP for logic-qubit entanglement. We first described the purification for both the bit-flip error and phase-flip error in the logic qubit. The entanglement purification for logic-qubit entanglement can be simplified to the purification of the physical-qubit entanglement, which can be performed in the next step. On the other hand, we also discussed the purification of the errors occurring in the physical-qubit entanglement. The bit-flip error on the physical qubit can be completely corrected locally. The phase-flip error occurs on the physical qubit equals to the bit-flip error on the logic qubit, which can also be well purified. Our EPP is suitable for the case that each logic qubit being arbitrary *N*-particle GHZ state. Our EPP may be useful for future long-distance quantum communication based on logic-qubit entanglement.

## Additional Information

**How to cite this article**: Zhou, L. and Sheng, Y.-B. Purification of Logic-Qubit Entanglement. *Sci. Rep.* **6**, 28813; doi: 10.1038/srep28813 (2016).

## References

[b1] Bennett C. H. . Teleporting an unknown quantum state via dual classical and Einstein-Podolsky-Rosen channels. Phys. Rev. Lett. 70, 1895 (1993). 10053414 10.1103/PhysRevLett.70.1895

[b2] Ekert A. K. Quantum cryptography based on Bell’s theorem. Phys. Rev. Lett. 67, 661 (1991). 10044956 10.1103/PhysRevLett.67.661

[b3] Long G. L. & Liu X. S. Theoretically efficient high-capacity quantum-keydistribution scheme. Phys. Rev. A 65, 032302 (2002).

[b4] Deng F.-G., Long G.-L. & Liu X.-S. Two-step quantum direct communication protocol using the Einstein-Podolsky-Rosen pair block. Phys. Rev. A 68, 042317 (2003).

[b5] Hu J. . Experimental quantum secure direct communication with single photons. Light Sci. Appl., 10.1038/lsa.2016.144 (2016). PMC6059926 30167186

[b6] Briegel H. J., Dür W., Cirac J. I. & Zoller P. Quantum repeaters: the role of imperfect local operations in quantum communication. Phys. Rev. Lett. 81, 5932 (1998).

[b7] Li T. & Deng F. G. Heralded high-efficiency quantum repeater with atomic ensembles assisted by faithful single-photon transmission. Sci. Rep. 5, 15610 (2015). 26502993 10.1038/srep15610 PMC4621506

[b8] Shi R. H., Mu Y., Zhong H., Cui J. & Zhang S. Two quantum protocols for oblivious set-member decision problem. Sci. Rep. 5, 15914 (2015). 26514668 10.1038/srep15914 PMC4626847

[b9] Wang T. Y., Cai X. Q., Ren Y. L. & Zhang R. L. Security of quantum digital signatures for classical messages. Sci. Rep. 5, 9231 (2015). 25782417 10.1038/srep09231 PMC4363884

[b10] He G. P. Security bound of cheat sensitive quantum bit commitment. Sci. Rep. 5, 9398 (2015). 25796977 10.1038/srep09398 PMC4369726

[b11] Chen B., Ma T. & Fei S. M. Entanglement detection using mutually unbiased measurements. Phys. Rev. A 30, 064302 (2014).

[b12] Chen X., Wang H. M., Ji D. T., Mu L. Z. & Fan H. Expected number of quantum channels in quantum networks. Sci. Rep. 5, 12128 (2015). 26173556 10.1038/srep12128 PMC4502510

[b13] Bennett C. H. . Purification of noisy entanglement and faithful teleportation via noisy channels. Phys. Rev. Lett. 76 722–725 (1996). 10061534 10.1103/PhysRevLett.76.722

[b14] Deutsch D. . Quantum privacy amplification and the security of quantum cryptography over noisy channels. Phys. Rev. Lett. 77, 2818–2821 (1996). 10062053 10.1103/PhysRevLett.77.2818

[b15] Dür W., Briegel H. J., Cirac J. I. & Zoller P. Quantum repeaters based on entanglement purification. Phys. Rev. A 59, 169 (1999).

[b16] Pan J. W., Simon C. & Zeilinger A. Entanglement purification for quantum communication. Nature 410, 1067–1070 (2001). 11323664 10.1038/35074041

[b17] Simon C. & Pan J. W. Polarization entanglement purification using spatial entanglement. Phys. Rev. Lett. 89, 257901 (2002). 12484922 10.1103/PhysRevLett.89.257901

[b18] Sheng Y. B., Deng F. G. & Zhou H. Y. Efficient polarization-entanglement purification based on parametric down-conversion sources with cross-Kerr nonlinearity. Phys. Rev. A 77, 042308 (2008).

[b19] Sheng Y. B. & Deng F. G. Deterministic entanglement purification and complete nonlocal Bell-state analysis with hyperentanglement. Phys. Rev. A 81, 032307 (2010).

[b20] Sheng Y. B. & Deng F. G. One-step deterministic polarization-entanglement purification using spatial entanglement. Phys. Rev. A 82, 044305 (2010).

[b21] Li X. H. Deterministic polarization-entanglement purification using spatial entanglement. Phys. Rev. A 82, 044304 (2010).

[b22] Deng F. G. One-step error correction for multipartite polarization entanglement. Phys. Rev. A 83, 062316 (2011).

[b23] Deng F. G. Efficient multipartite entanglement purification with the entanglement link from a subspace. Phys. Rev. A 84, 052312 (2011).

[b24] Sheng Y. B., Zhou L. & Long G. L. Hybrid entanglement purification for quantum repeaters. Phys. Rev. A 88, 022302 (2013).

[b25] Zwerger M., Briegel H. J. & Dür W. Universal and optimal error thresholds for measurement-based entanglement purification. Phys. Rev. Lett. 110, 260503 (2013). 23848858 10.1103/PhysRevLett.110.260503

[b26] Zwerger M., Briegel H. J. & Dür W. Robust of hashing protocols for entanglement purification. Phys. Rev. A 90, 012314 (2014).

[b27] Ren B. C., Du F. F. & Deng F. G. Two-step hyperentanglement purification with the quantum-state-joining method. Phys. Rev. A 90, 052309 (2014).

[b28] Sheng Y. B. & Zhou L. Deterministic polarization entanglement purification using time-bin entanglement. Laser Phys. Lett. 11, 085203 (2014).

[b29] Sheng Y. B. & Zhou L. Deterministic entanglement distillation for secure double-server blind quantum computation. Sci. Rep. 5, 7815 (2015). 25588565 10.1038/srep07815 PMC4295105

[b30] Wang C., Zhang Y. & Jin G. S. Entanglement purification and concentration of electron-spin entangled states using quantum-dot spins in optical microcavities. Phys. Rev. A 84, 032307 (2011).

[b31] Wang C., Zhang Y. & Zhang R. Entanglement purification based on hybrid entangled state using quantum-dot and microcavity coupled system. Opt. Express 19, 25685–25695 (2011). 22273961 10.1364/OE.19.025685

[b32] Gonta D. & van Loock P. Dynamical entanglement purification using chains of atoms and optical cavities. Phys. Rev. A 84, 042303 (2011).

[b33] Gonta D. & van Loock P. High-fidelity entanglement purification using chains of atoms and optical cavities. Phys. Rev. A 86, 052312 (2012).

[b34] Martín-Delgado M. A. & Navascués M. Single-step distillation protocol with generalized beam splitters. Phys. Rev. A 68, 012322 (2003).

[b35] Bombin H. & Martin-Delgado M. A. Entanglement distillation protocols and number theory. Phys. Rev. A 72, 032313 (2005).

[b36] Yang M., Song W. & Cao Z. L. Entanglement purification for arbitrary unknown ionic states via linear optics. Phys. Rev. A 71, 012308 (2005).

[b37] Fröwis F. & Dür W. Stable macroscopic quantum superpositions. Phys. Rev. Lett. 106, 110402 (2011). 21469844 10.1103/PhysRevLett.106.110402

[b38] Fröwis F. & Dür W. Stability of encoded macroscopic quantum superpositions. Phys. Rev. A 85, 052329 (2012). 10.1103/PhysRevLett.106.110402 21469844

[b39] Kesting F., Fröwis F. & Dür W. Effective noise channels for encoded quantum systems. Phys. Rev. A 88, 042305 (2013).

[b40] Dür W., Skotinioti M., Fröwis F. & Kraus B. Improved quantum metrology using quantum error correction. Phys. Rev. Lett. 112, 080801 (2014).

[b41] Ding D., Yan F. L. & Gao T. Preparation of km-photon concatenated Greenberger-Horne-Zeilinger states for observing distinctive quantum effects at macroscopic scales. J. Opt. Soc. Am. B 30, 3075–3078 (2013).

[b42] Lu H. . Experimental realization of a concatenated Greenberger-Horne-Zeilinger state for macroscopic quantum superpositions. Nat. Photon. 8, 364–368 (2014).

[b43] Sheng Y. B. & Zhou L. Entanglement analysis for macroscopic Schrödinger’s Cat state. EPL 109, 40009 (2015).

[b44] Sheng Y. B. & Zhou L. Two-step complete polarization logic Bell-state analysis. Sci. Rep. 5, 13453 (2015). 26307327 10.1038/srep13453 PMC4549687

[b45] Zhou L. & Sheng Y. B. Complete logic Bell-state analysis assisted with photonic Faraday rotation. Phys. Rev. A 92, 042314 (2015).

[b46] Zhou L. & Sheng Y. B. Feasible logic Bell-state analysis with linear optics. Sci. Rep. 6, 20901 (2016). 26877208 10.1038/srep20901 PMC4753447

[b47] Qu C. C., Zhou L. & Sheng Y. B. Entanglement concentration for concatenated Greenberger-Horne-Zeilinger state. Quantum Inf. Process. 14, 4131–4146 (2015).

[b48] Pan J. . Efficient entanglement concentration for concatenated Greenberger-Horne-Zeilinger state with the cross-Kerr nonlinearity. Quant. Inf. Process. 15, 1669–1687 (2016).

[b49] Nemoto K. & Munro W. J. Nearly deterministic linear Optical controlled-NOT gate. Phys. Rev. Lett. 93, 250502 (2004). 15697884 10.1103/PhysRevLett.93.250502

[b50] Lin Q. & Li J. Quantum control gates with weak cross-Kerr nonlinearity. Phys. Rev. A 79, 022301 (2009).

[b51] Wei H. R. & Deng F. G. Scalable photonic quantum computing assisted by quantum-dot spin in double-sided optical microcavity. Opt. Express 21, 17671–17685 (2013). 23938640 10.1364/OE.21.017671

[b52] Beenakker C. W. J., DiVincenzo D. P., Emary C. & Kindermann M. Charge detection enables free-electron quantum computation. Phys. Rev. Lett. 93, 020501 (2004). 15323887 10.1103/PhysRevLett.93.020501

[b53] Wei H. R. & Deng F. G. Compact quantum gates on electron-spin qubits assisted by diamond nitrogen-vacancy centers inside cavities. Phys. Rev. A 88, 042323 (2013).

[b54] Rong X. . Experimental fault-tolerant universal quantum gates with solid-state spins under ambient conditions. Nat. Commun. 6, 8748 (2015). 26602456 10.1038/ncomms9748 PMC4674779

